# Study of the normal heart size in Northwest part of Iranian population: a cadaveric study

**DOI:** 10.15171/jcvtr.2016.25

**Published:** 2016-09-26

**Authors:** Shabnam Mohammadi, Arya Hedjazi, Maryam Sajjadian, Naser Ghoroubi, Maryam Mohammadi, Saeed Erfani

**Affiliations:** ^1^Microanatomy Research Center, Mashhad University of Medical Sciences, Mashhad, Iran; ^2^Department of Anatomy and Cell Biology, School of Medicine, Mashhad University of Medical Sciences, Mashhad, Iran; ^3^Legal Medicine Research Center, Legal Medicine Organization, Tehran, Iran; ^4^Department of Public Health, School of Health, Shahid Beheshti University of Medical Sciences, Tehran, Iran; ^5^Neurogenic Inflammation Research Center, Mashhad University of Medical Sciences, Mashhad, Iran

**Keywords:** Human Heart, Cadaver, Gross Anatomy, Morphometry

## Abstract

***Introduction:*** The heart is in a muscular organ in the middle mediastinum. According to our knowledge, there is no standard data about the anthropologic parameters of normal Iranian hearts. Hence, the aim of the present study was to investigate the normal heart size in Iranian cadavers.

***Methods: ***In a cross-sectional study, 550 cadavers (104 female/446 male) from June 2014 to July 2015 in the Razavi Khorasan province of Iran were included in the study. After approval of the Ethical Committee, cadavers were divided into 10 groups based on age groups. Length, width, weight, chordae tendineae, papillary muscles, and heart valves were measured using vernier caliper. Finally, data were analyzed using SPSS software.

***Results: ***The mean values of the demographic data were as follows: age= 42.12 ± 21.34 years; weight = 60.38 ± 15.32 kg; height = 158.14 ± 23.77 cm; and BMI = 24.66 ± 17.60 kg/m^2^. The mean values of the heart length, width, chordae tendineae, pupillary muscles, weight, and index of the heart were 11.41 ± 2.15 cm, 8.21 ± 4.38 cm, 19.41 ± 6.70, 5.74 ± 1.96, 247.78 ± 62.27 grams, and 5.74 ± 1.96, respectively. In addition, the circumference of the tricuspid valve, circumference of the mitral valves, and tricuspid and mitral areas were 8.80 ± 1.11 cm, 9.43 ± 1.44 cm, 4.11 ± 0.71 cm^2^, and 4.50 ± 0.90 cm^2^, respectively.

***Conclusion:*** Mean values of the heart’s length and width was similar to previous reports from western population. The circumference of the tricuspid valve was less than the textbook’s data, while circumference of the mitral valves was more than it. The study findings provide valuable information about standard data of the heart in the Iranian population, which is useful for surgeons as well as anthropologists. However, multi-center studies with a larger sample size are required to complete data about anatomical characteristics of normal hearts.

## Introduction

Several factors such as race, age, gender, physical activity, nutrition, and health status affect the size of organs such as the heart.^1^ There are vast differences in body organs among races, as well as ethnic and nationality groups.^1^ In some races, heart weights increased with age but decreased with age in other races.^2-10^ In most races, the dimension of the heart was larger in men than women.^2-4,9,11-13^ Exercise and physical activity cause a reduction in fat tissue of the heart, but increases the muscle size of the heart.^1^ Malnutrition, especially in growth periods causes a reduction in the growth of organs.^1^ Disease is also another factor that may change the size and anatomy of organs.^1^

Many heart diseases such as regurgitation, stenosis, and prolapse cause damage to heart valves.^14^

Severe damage such as endocarditis, dysplastic valve pathology, and rheumatic valve diseases necessitate the replacement of valves and for the manufacture of valves, it is important to know the exact anatomy of the heart.^14^

Papillary muscles and tendinous cords are short and thick in congenital malformation of the tricuspid valve.^15^ Hence, knowing the anatomic variation of papillary muscles is important in papillotomy and commissurotomy in surgical resection of the valves, as well as in cases of rheumatic and traumatic damages.^15^

According to *Gray’s Anatomy*, the heart length, width, and thickness are 12 cm, 8.5 cm, and 6 cm, respectively.^16^ In addition, the mean weight of the heart is 280-340 g in males and 230-280 g in females.^16^ The mean circumference of the mitral valve is 9 cm in males and 7.2 cm in females, whereas for the tricuspid valve it is 10.8 cm in females and 11.4 cm in males.^16^ The mean heart weight has reported to range from 248 to 345 g in men and 164 to 299 g in women in Asian populations.^2,4,9,10^ This population also has 0 to10, papillary muscles.^15,17,18^ The mitral area is 8.8 cm in Japanese and 7-10 cm in Indians.^14,19-22^ The annular circumference of the tricuspid leaflet is 85.95 to 107.5 mm in India.^23^

No data exists about the standard dimensions of the normal heart in Iranian populations. Hence, the objective of this study was to evaluate the standard size of the normal heart among Iranian cadavers.

## Materials and Methods

We performed a cross-sectional study on 550 cadavers (104 female, 446 male), referred to the dissection hall of the Forensic Medicine Organization, Razavi Khorasan province, from June 2014 to July 2015.

Inclusion criteria were as follows: Fresh Iranian cadavers with no history of poisoning, alcohol, smoking, drug abuse, or hypertension; and no evidence of trauma or abnormality of the heart. The cause of death was sharp and blunt trauma including car accident. Cadavers who had died from unknown causes or who had cardiac diseases were excluded from the study.

Demographic data, including gender, age, and body weight and height, were recorded (Table 1). The index was calculated as heart weight divided by body weight. Body mass index (BMI) was also calculated as weight (kg)/height (m^2^). Cadavers’ race was Persian. There is living place of cadavers in Table 2.

**Table 1:** Demographic data of Iranian cadavers (N=550) in Razavi Khorasan province, Iran

**Age groups**	**Age (years)**	**Gender (female/male)**	**Height (cm)**	**Weight (kg)**
<10	2.90±2.55	11/24	89.65±34.74	13.51±16.31
10-19	16.21±2.53	12/30	159.54±14.28	58.85±14.42
20-29	24.68±2.82	18/72	163.08±11.76	63.94±7.44
30-39	34.21±2.75	13/77	162.42±17.98	65.32±6.70
40-49	44.38±2.57	18/89	162.48±13.12	62.96±8.65
50-59	54.10±2.74	11/46	162.14±16.32	63.07±9.39
60-69	63.88±2.29	8/55	165.46±4.58	66.38±6.48
70-79	74.70±2.48	9/31	163.15±14.73	62.55±6.41
80-89	84.00±2.99	2/16	163.94±5.43	64.16±6.46
90-99	90.62±1.06	2/6	164.12±2.35	57.87±7.93

Values are presented as mean± SD or number.

**Table 2:** The living place of Iranian cadavers in Iran (N=550)

**City**	**N0. of cadavers**	**City**	**No. of cadavers**	**City**	**No. of cadavers**
Mashhad	442	Khaf	3	Shirvan	1
Neyshabur	11	Dargaz	3	Gonabad	1
Torbat-e Jam	10	Zahedan	3	Isfahan	1
Chenaran	10	Ahvaz	2	Shahrood	1
Sabzevar	8	Ilam	2	Borujerd	1
Fariman	7	Tabas	2	Hamedan	1
Bojnourd	6	Qaen	2	Kashmar	1
Tehran	6	Zaveh	2	Zabol	1
Ghochan	6	Minoodasht	2	Birjand	1
Torbat-e Heydarieh	5	Gonbad	1	Bajestan	1
Taybad	3	Gorgan	1	Bardaskan	1

Five hundred fifty cadavers were divided into 10 different age groups: Group A (0-9 years), Group B (10-19 years), Group C (20-29 years), Group D (30-39 years), Group E (40-49 years), Group F (50- 59 years), Group G (60-69 years), Group H (70-79 years), Group I (80-89 years), and Group J (90-99 years).

The thorax was opened by a midline incision, and the heart was washed with tap water. The length of the heart was measured from the base to the apex using a vernier caliper. Caliper calibration performed previously based on ISO guidelines. The greatest distance between the anterior and posterior surfaces of the heart was considered to be its thickness. The heart’s weight was also measured with the help of an electronic weighing machine (Pand Azma 3100, Iran). The number of pupillary muscles and chordae tendina were recorded. The Circumference and area of tricuspid and mitral valves were measured using Image J software. Measurements for all cadavers were performed by an expert anatomist. Photographs were taken using a Canon digital camera.

### Statistical analysis

Data were expressed as mean ± standard deviations and were analyzed using SPSS 20.0 software. *P* values less than 0.05 were considered significant. The normality of data was assessed using the Kolmogorov-Smirnov test. The correlation between anthropometric values and morphometric data of the heart was investigated using the Pearson correlation. Comparisons between groups were carried out based on independent sample *t* tests (for two groups) and analysis of variance (for more than two groups).

## Results

Demographic characteristics of cadavers are summarized in Table 1. Five hundred fifty Iranian cadavers (104 females/446 males) with a mean age of 42.12 ± 21.34 years were enrolled to the study (Table 2). The values obtained for height ranged between 48 and 182 cm, with an average of 158.14 cm. The weight of the cadavers ranged from 2.5 to 86 g, with an average of 60.38 g. The mean body mass index was 24.66 ± 17.60 kg/m^2^.

The mean length of the heart was 11.41 cm (range, 3 to 14 cm). The average width of the heart measured 8.21 cm. The minimum weight of the heart was 21 g, and its maximum weight was 593 g. The chordae tendineae ranged between 4 and 35, with a mean of 19.41 ± 6.70. The mean number of pupillary muscles was 5.74 ± 1.96 (range, 1 to 10). The index of the heart varied from 0.5 to 75, with a mean value of 5.09.

**Figure 1:**
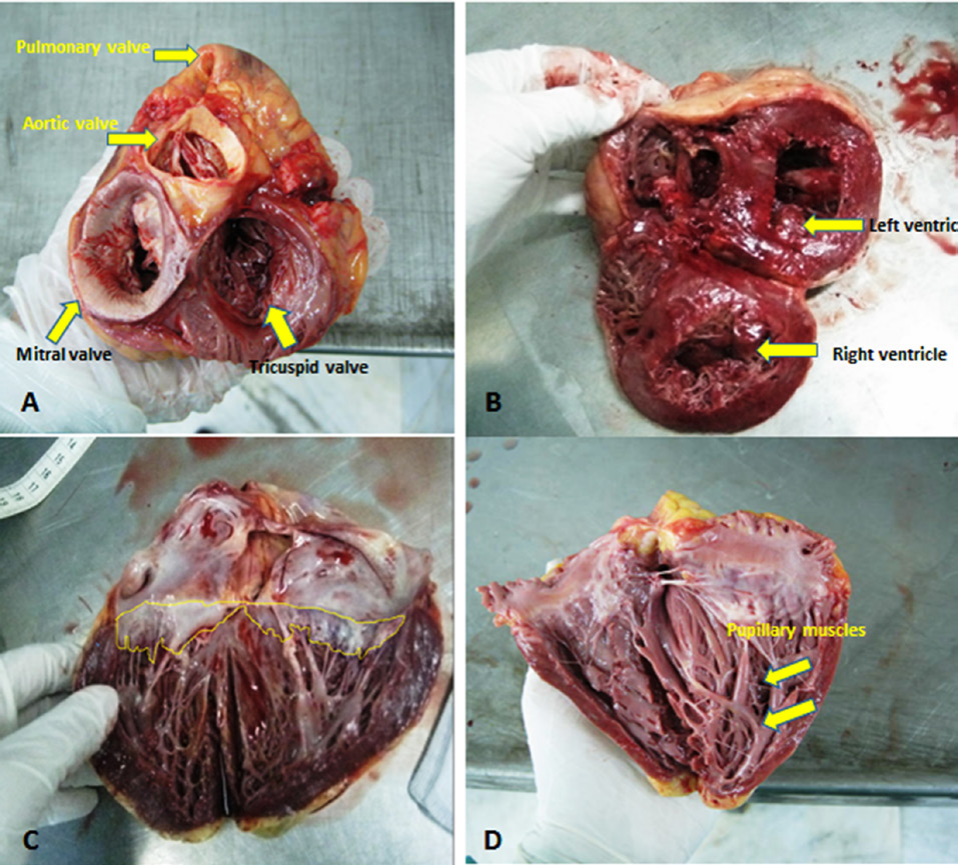
(A) The four valves of the heart; (B) Transvers section of the ventricles; (C) Mitral Valve opened to measure the area of anterior and posterior leaflets; (D) Arrows show papillary muscles.

The annular circumference of the tricuspid valve and mitral valve was 8.80 ± 1.11 and 9.43 ± 1.44 cm, respectively. In addition, the mean value of the tricuspid area was 4.11 ± 0.71 cm^2^, while this value was 4.50 ± 0.90 cm^2^ for the mitral area (Figure 1).

As shown in Table 3, the longest hearts were observed in groups D and F, while the shortest was in group A. The smallest width of the heart was seen in cadavers 0-9 years old, while the greatest width of the heart was found in cadavers 70-79 years old. The weight of the heart was heaviest in the ninth decade, while it was lightest in the first of life. The index of the heart was the largest in group A, and least in group G.

**Table 3:** Length, width, chordae tendineae, pupillary muscle, weight, and index of the heart of Iranian cadavers in different age groups

**Age groups**	**Length (cm)**	**Width (cm)**	**No. of Chordae tendineae **	**No. of Pupillary muscle**	**Weight (g)**	**Index**
<10	7.25±3.25	5.64±2.62	14.37±6.01	4.62±2.31	142.48±100.62	18.24±16.66
10-19	11.79±1.93*	8.22±1.59	20.69±6.91***	5.92±1.86	253.38±70.64*	4.64±1.96
20-29	11.60±1.89*	8.04±1.63	18.62±6.10**	5.73±1.83	249.60±48.61*	3.95±0.92
30-39	11.89±1.60*	8.98±7.17#	19.52±6.33##	5.88±1.81**	261.18±61.23*	4.03±0.99
40-49	11.73±1.44*	8.12±1.32	19.21±6.82#	5.53±2.04	255.43±42.79*	4.53±4.82
50-59	11.89±1.61*	8.23±1.53	20.94±7.24*	5.98±1.75**	254.64±55.73*	4.41±3.14
60-69	11.76±1.77*	8.18±1.63	20.73±6.50*	6.34±1.95***	247.15±45.44*	3.74±0.73
70-79	11.47±1.56*	9.65±10.68##	21.12±5.96*	5.80±1.92	259.85±31.21*	4.19±0.64
80-89	10.94±2.41*	8.08±2.02	17.83±7.98	5.16±2.45	263.27±61.41*	4.13±0.93
90-99	10.37±2.87***	7.43±2.19	18.87±4.76	5.50±1.51	266.37±38.30*	4.67±0.87

Values are expressed as mean ± SD. Comparison between groups was made using ANOVA and Tukey test. * *P* = 0.000 compared to group A within column, ** *P* = 0.04 compared to group A within column, *** *P* = 0.001 compared to group A within column, # *P* = 0.005 compared to group A within column, ## *P* = 0.003 compared to group A within column.

Table 4 shows the characteristics of the mitral and tricuspid valves in the hearts of cadavers in different age groups. A significant difference was evident in the values of the mitral and tricuspid valves between group A (0–9 years old) and all other groups (*P *< 0.05). The same result was found for length, width, chordae tendineae, pupillary muscle, weight, and index of the Iranian hearts (*P *< 0.05).

**Table 4:** Characteristics of the mitral and tricuspid valves in the heart of Iranian cadavers in different age groups

**Age groups**	**Circumference of the tricuspid valve (cm)**	**Tricuspid anterior area (cm** ^2^ **)**	**Tricuspid septal area (cm** ^2^ **)**	**Tricuspid posterior area (cm** ^2^ **)**	**Circumference of the mitral valve (cm)**	**Mitral anterior area (cm** ^2^ **)**	**Mitral posterior area (cm** ^2^ **)**
<10	7.01±1.80	1.00±0.49	0.89±0.49	1.02±0.63	6.39±2.59	1.78±1.26	0.80±0.48
10-19	8.89±0.86*	1.34±0.42#	1.13±0.33	1.69±0.30*	9.63±1.29*	3.49±0.80*	1.13±0.41#
20-29	8.91±0.75*	1.26±0.38	1.14±0.32	1.78±0.30*	9.68±0.88*	3.53±0.44*	1.13±0.40*
30-39	9.05±0.89*	1.30±0.39#	1.16±0.40#	1.81±0.28*	9.73±0.94*	3.53±0.60*	1.17±0.39*
40-49	8.84±1.16*	1.22±0.44	1.14±0.41***	1.76±0.40*	9.54±1.28*	3.42±0.65*	1.15±0.39*
50-59	8.79±0.85*	1.25±0.41	1.15±0.40	1.68±0.31*	9.68±1.27*	3.50±0.72*	1.14±0.40**
60-69	9.03±0.95*	1.27±0.42	1.15±0.32	1.82±0.30*	9.57±0.81*	3.50±0.43*	1.09±0.36***
70-79	8.94±0.88*	1.17±0.45	1.22±0.39#	1.79±0.29*	9.56±0.90*	3.47±0.55*	1.11±0.42***
80-89	9.25±0.90*	1.50±0.31**	1.03±0.39	1.79±0.21*	9.72±0.92*	3.56±0.49*	1.12±0.38
90-99	8.47±0.60*	1.13±0.34	1.03±0.29	1.76±0.17*	9.73±0.53*	3.28±0.25*	1.41±0.34*

Values are expressed as mean ± SD. Comparison between groups was made using ANOVA and Tukey test. #*P *= 0.01 compared to group A (0-9 years old), **P = 0.002 compared to group A, *P = 0.03 compared to group A, *P = 0.000 compared to group A.

The posterior cusp of the mitral valve was significantly smaller than the anterior cusp of this valve. The septal cusp of the tricuspid was remarkably smaller than the anterior or posterior cusp of the tricuspid valve (Tables 4 and 5). The circumference and area of the mitral valve were significantly higher in males than females (Table 6). No significant differences were evident in other morphometric parameters of the heart between females and males (*P *> 0.05). Table 7 shows a strong correlation between the anthropometric values of the cadavers and the morphometric data of the heart (*P *< 0.05).

**Table 5:** Mediolateral and anteroposterir diameters of the mitral and tricuspid valves in the heart of Iranian cadavers in different age groups

**Age groups**	**Mediolateral diameter of tricuspid valve (cm)**	**Anteroposterior diameter of tricuspid valve (cm)**	**Mediolateral diameter of Mitral valve (cm)**	**Anteroposterior diameter of Mitral valve (cm)**
<10	1.58±0.19	2.23±0.57	1.53±0.28	2.03±0.82
10-19	1.81±0.13*	2.80±0.26*	1.94±0.17*	3.07±0.41*
20-29	1.82±0.12*	2.84±0.24*	1.94±0.14*	3.10±0.37*
30-39	1.84±0.14*	2.87±0.28*	1.95±0.14*	3.10±0.29*
40-49	1.81±0.15*	2.81±0.37*	1.92±0.17*	3.04±0.40*
50-59	1.80±0.13*	2.80±0.26*	1.95±0.16*	3.08±0.40*
60-69	1.83±0.15*	2.87±0.30*	1.92±0.12*	3.05±0.26*
70-79	1.82±0.14*	2.84±0.28*	1.89±0.20*	3.04±0.28*
80-89	1.87±0.14*	2.94±0.28*	1.95±0.14*	3.13±0.29*
90-99	1.75±0.09	2.70±0.19	1.95±0.08*	3.10±0.17*

Values are expressed as mean± SD. Comparison between groups was made using ANOVA and Tukey test. *P=0.000 compared to group A within column

**Table 6:** Length, width, chordae tendineae, weight, pupillary muscles, and index of the heart of Iranian cadavers of different genders

**Morphometric characterizes**	**Gender**		*** P*** ** value**
	**Female**	**Male**	
Length (cm)	11.26±2.35	11.44±2.11	0.52
Width (cm)	7.93±1.84	8.28±4.79	0.63
Chordae tendineae	19.23±6.85	19.45±6.67	0.79
Weight (g)	242.74±73.02	248.96±59.52	0.12
Pupillary muscles	5.48±1.93	5.80±1.96	0.79
Index	5.77±6.21	4.94±5.86	0.12
Circumference of the tricuspid valve (cm)	8.72±1.28	8.82±1.06	0.37
Tricuspid area (cm^2^)	4.06±0.84	4.12±0.67	0.22
Circumference of the mitral valve (cm)	9.19±1.78	9.49±1.35	0.01
Mitral area (cm^2^)	4.36±1.09	4.54±0.85	0.02

Values are presented as mean ± SD. Independent samples *t* test was used to compare values.

**Table 7:** Correlation (r) between morphological parameters of heart and demographic characteristics

**Morphological parameters**		**Age**	**Height**	**Body weight**	**BMI**
Length (cm)	Pearson correlation	0.191	0.414	0.411	0.028
	Sig. (2-tailed)	P=0.000	P=0.000	P=0.000	P>0.05
Width (cm)	Pearson correlation	0.94	0.115	0.120	0.022
	Sig. (2-tailed)	P=0.02	P=0.007	P=0.005	P>0.05
Corda tendinae Number of	Pearson correlation	0.136	0.177	0.181	0.061
	Sig. (2-tailed)	P=0.001	P=0.000	P=0.000	P>0.05
Number of Pupillary muscles	Pearson correlation	0.081	0.166	0.149	0.033
	Sig. (2-tailed)	P>0.05	P=0.000	P=0.000	P>0.05
Weight (gram)	Pearson correlation	0.236	0.351	0.393	0.057
	Sig. (2-tailed)	P=0.000	P=0.000	P=0.000	P>0.05
Index	Pearson correlation	-0.284	-0.604	-0.662	-0.151
	Sig. (2-tailed)	P=0.000	P=0.000	P=0.000	P=0.000
Circumference of the tricuspid valve (cm)	Pearson correlation	0.219	0.355	0.370	0.094
	Sig. (2-tailed)	P=0.000	P=0.000	P=0.000	P=0.02
Tricuspid area (cm^2^)	Pearson correlation	0.215	0.382	0.342	-0.453
	Sig. (2-tailed)	P=0.000	P=0.000	P=0.000	P=0.000
Circumference of the mitral valve (cm)	Pearson correlation	0.255	0.481	0.467	0.095
	Sig. (2-tailed)	P=0.000	P=0.000	P=0.000	P=0.02
Mitral area (cm^2^)	Pearson correlation	0.256	0.472	0.463	-0.497
	Sig. (2-tailed)	P=0.000	P=0.000	P=0.000	P=0.000

Correlations were assessed using Pearson correlation coefficients.

## Discussion

Mean values of the heart’s length, width, and thickness in *Gray’s Anatomy* were 12 cm, 8.5 cm, and 6 cm, respectively.^16^ Our findings are similar to the textbook’s data, as the mean length of a normal heart was 11.41 cm and the mean width of a normal heart measured 8.21 cm. In a study in Bangladesh, the mean length of hearts was 10.5 cm in males and 9.2 cm in females, and the mean width was 8.51 cm in males and 7.67 cm in females.^13^ A positive relationship exists between demographic values and heart dimensions, which is similar to our findings.^8^

Based on the standard textbook of anatomy, the mean weight of the heart is 280-340 g in men and 230-280 g in women.^16^ In the present study, the mean weight of the heart was 242.74 g in females and 248.96 g in males. These differences might be due to the geography or race of specimens studied.

The mean heart weight is higher in males than females in American and Caucasian population.^2,4,6,8-10^ Furthermore, and similar to our findings, heart weight significantly correlates with age, weight, and height.^2-4,8,9^ In the Japanese population, heart weight increases with age up to 90 years of age and then decreases afterwards.^7^ In the Chinese population, heart weight is constant and may even increase with age.^10^

The mean heart weight in Thailand is reported to range between 291 and 302 g in men and 246 and 259 g in women.^3,12^ In the Korean population, heart weight ranged between 305 and 345 g in males and 265 and 299 g in females.^4,9^ A study was conducted on 109 Indian cadavers (86 males and 23 females) with a mean age of 15-78 years for males and 1-55 years for females. Their results showed that heart weight was 270.28 g in males and 204.35 g in females. Heart weight is directly related to body weight and decreases with increase in age.^2^ In another study conducted using Bangladesh adults, heart weight was 247.92 g in men and 164.29 g in women.^13^

The chordae tendineae ranges between 1 and 10 in Brazil, 3 and 22 in Indian cadavers,^24^ and 4 and 35 in the present study.

It is also important to be aware of anatomic variations of the papillary muscles for mitral valve replacement, papillotomy, and commissurotomy.^18^ The papillary muscles range between 3 and 10 in the United States,^25^ 0 and 10 in Asian populations,^15,17,18^ and 1 and 10 in our samples. In India, the number of anterior and posterior papillary muscles in the left ventricle of 96 fixed cadavers was between 2-10. The mean length of the anterior papillary muscle was 1.49, width 0.82 and thickness was 0.64 cm. The mean length of the posterior papillary muscle was 1.05, width 0.63 while thickness was 0.5 cm.^8^ Variations in papillary muscles were evaluated in the right ventricles of 400 Turkish adult cadavers. The results showed that in cases with cardiac-related disease as the cause of death, papillary muscles were low or nonexistent, while the papillary muscles in cadavers with non-cardiac cause of death was 100% of the cases or more.^26^

An understanding of the measurements of the human valves may be of benefit for designing and manufacturing a support house.^21^ According to *Gray’s Anatomy*, the average circumference of the mitral valve is 9 cm in males and 7.2 cm in females.^16^

The mitral area ranges between 6.4 and 8.2 cm^2^ in the Unites States based on a two-dimensional echocardiogram.^27^ In Brazil, Andrade et al reported the annular circumference of mitral and tricuspid valves to be 9.3 cm based on MATLAB software, and the mean values of the mitral and tricuspid areas were 4.72 cm and 6.28 cm, respectively.^28^

In an African study, the circumference of the mitral valve was 10.1 cm, the mean length of the free edge of the mitral valve was 9.1 cm, and the mean lengths of the free edge of the anterior and posterior cusps were 2.7 cm and 1.3 cm, respectively.^29^

The annular circumference is 8.8 in Japanese,^30^ while it ranges between 7 and 10 cm in the Indian population.^14,15,28,31,32^ The area of the anterior valve is 0.8 compared to 2 of the posterior valve.^14,15,28,31,32^

In Indian autopsies, measurement of the mitral valve showed that the area of the anterior leaflet was 1.6 cm larger than the posterior leaflet. In addition, the circumference of the mitral valve was 8.24 cm.^15^ Another study by Gupta and colleagues in India, showed that the average circumference of the mitral valve was 9.11 cm. The average length of the free edge of the anterior leaflet was 5.6 cm while the posterior leaflet was 8.89 cm. The anterior and posterior leaflets were 3.32 cm^2^ and 4.14 cm^2^ in area, respectively.^14^ Our finding showed the annular circumference of the mitral valve to be 9.43 cm and the mitral area to be 4.50 cm^2^.

In the Western textbook (*Gray’s Anatomy*, 39th edition), the mean value of the tricuspid circumference was noted to be 10.8 cm in females and 11.4 cm in males.^16^ In an autopsy study by Kitzman et al in the United States, the annular circumference of valves was higher in males than females.^6^ Similarly, in another autopsy study in China, the annular circumference of valves was found to be higher in males than females.^10^ In a study in Poland, the annular circumference of the tricuspid valve was 112.97 mm in men and 112.17 mm in women.^33^ In another cadaveric study including 100 formalin fixed hearts aged 8 to 85 years, the tricuspid circumference ranged from 89.34 to 107.5 mm in males and 85.95 to 104 mm in females.^23^

In the present study, the annular circumferences of the tricuspid valve and the tricuspid area were 8.80 cm and 4.11 cm^2^, respectively. In a Brazilian study on fetuses, the mean values of the mitral and tricuspid areas were 84.06 mm^2^ and 84.49 mm^2^. ^34^ Kishore and colleagues reported that the annular circumference of the tricuspid valve was 17.39 mm in fetuses less than 30 weeks old compared to 47.66 mm in fetuses older than 30 weeks.^35^ In the present study, the annular circumference of the tricuspid valve and tricuspid area in fetuses was 58.44 mm and 21.39 mm^2^, respectively. This study is the first to investigate standard data of Iranian normal hearts. Having standard data on the heart is useful for surgeons as well as anthropologists. The findings of the present study can provide valuable data in the standardization of the anthropologic values of the heart in the Iranian population. However, multi-center studies with a larger sample size are required to complete data about anatomical characteristics of normal hearts.

## Limitations

Our limitation in this study was that most of our sample was males, which may result in some sampling bias.

## Ethical issues

This research was approved by the Ethics Research Committee of Mashhad Legal Medicine Organization (ethical code: 93034). All experiments were performed in compliance with the laws for human trial.

## Competing interests

None.

## Acknowledgments

This study was financially supported by a research grant (number 93034) from Research Council of Mashhad Legal Medicine Organization. The author would like to thank the Mashhad Legal Medicine Organization for their help and financial support.
